# Visit-to-visit blood pressure variability is a marker of cardiac diastolic function and carotid atherosclerosis

**DOI:** 10.1186/1471-2261-14-188

**Published:** 2014-12-15

**Authors:** Rieko Okada, Akira Okada, Takashi Okada, Mamoru Nanasato, Kenji Wakai

**Affiliations:** Department of Preventive Medicine, Nagoya University Graduate School of Medicine, 65 Tsurumai-cho, Showa-ku, Nagoya 466-8550 Japan; Okada Medical Clinic, Nagoya, Japan; Cardiovascular Center, Nagoya Daini Red Cross Hospital, Nagoya, Japan

## Abstract

**Background:**

The associations between visit-to-visit blood pressure (BP) variability and cardiac function and carotid atherosclerosis is not clear.

**Methods:**

Study subjects were 144 subjects (80 were female, aged 73 ± 9 years) who underwent echocardiography and cervical ultrasonography. The ratio of early ventricular filling velocity to early diastolic mitral annular velocity (E/e’), ejection fraction, left ventricular mass index (LVMI), and maximum intima-media thickness (max-IMT) of the carotid artery were compared between the highest (high variability) and lowest (low variability) tertiles of the standard deviation of systolic BP (9.9 ± 3.5 mmHg).

**Results:**

E/e’ and max-IMT were significantly greater in the high variability group than in the low variability group after adjusting for age, sex, baseline systolic BP, and other covariates (high variability vs. low variability; E/e’: 13.03 ± 5.33 vs. 10.66 ± 3.30, multivariate-adjusted difference (β) = 1.82, 95% confidence interval 0.06–3.58; max-IMT: 1.65 ± 0.43 mm vs. 1.42 ± 0.46 mm, β = 0.20 mm, 95% confidence interval 0.03–0.36 mm). There were no significant differences in LVMI or ejection fraction.

**Conclusion:**

These results indicate that high visit-to-visit BP variability is associated with diastolic function and carotid atherosclerosis, and is a possible risk factor for diastolic dysfunction and atherosclerosis.

## Background

Blood pressure (BP) variability is now considered to be an important risk factor for cardiovascular diseases (CVD) [1]. Visit-to-visit BP variability, which is assessed in several clinical visits and reflects long-term BP variability, was recently reported to be a strong predictor of CVD morbidity and mortality independently of BP level [2–4]. Carotid atherosclerosis, a marker for the risk of CVD [5], may also be associated with visit-to-visit BP variability [6]. However, there are limited data regarding the association between visit-to-visit BP variability and cardiac function measures [7]; thus, the mechanism underscoring the association between BP variability and CVD is unclear. Therefore, this study was conducted to investigate whether visit-to-visit BP variability is associated with measures of cardiac function and carotid atherosclerosis in subjects who underwent echocardiography and cervical ultrasonography at the same time.

## Methods

### Study subjects

This study was performed in 144 consecutive subjects (64 were male and 80 were female; mean age 73 years [SD, 9 years], range 35–93 years) who underwent echocardiography and cervical ultrasonography at the same time between September 2011 and August 2013 at Okada Medical Clinic in Nagoya, Japan. These data were collected during routine care and were extracted from medical charts, thus the patients for the examinations were not randomly collected for this study. The study subjects were; 15 with the history of coronary heart diseases, 10 with the history of stroke, 42 with diabetes, 118 with hypertension, and 46 with hyperlipidemia. Systolic and diastolic BP, heart rate, height, and weight at the time of echocardiography were used as baseline variables. Blood samples were also taken in the morning after an 8-h overnight fast to measure laboratory variables. Fasting plasma glucose, hemoglobin A1c, and serum low-density lipoprotein cholesterol, uric acid and creatinine were measured using standard laboratory techniques, and the estimated glomerular filtration rate was calculated as previously described [8]. We also recorded whether the subjects used calcium-channel blockers, angiotensin receptor blockers, β-blockers, glucose-lowering drugs, or lipid-lowering drugs. Three subjects were taking low-dose diuretics for their symptom of heart failure. Histories of CVD (coronary heart diseases or stroke) were also recorded. Subjects with atrial fibrillation or valvular diseases were excluded from the study. The study was approved by the ethics committee of Nagoya University Graduate School of Medicine (approval number 2013–0182) and the ethics committee of Nagoya Medical Association.

### BP measurement and BP variability

We retrospectively reviewed the subjects’ medical records to retrieve BP values recorded over 1 year before the echocardiographic examination. BP was measured in an outpatient clinic every 1 to 3 months using the conventional cuff method using the standard protocol of BP measurement by the Japanese Society of Cardiovascular Disease Prevention. BP variability was retrospectively assessed. The standard deviation (SD) of systolic BP from 4 to 13 BP measurements was used as an index of BP variability, as reported in earlier studies [2, 3, 6, 9–11]. Subjects were classified into tertiles of the SD of systolic BP as used in the previous study [2], because the number of study subjects was not enough to use quartile [9] or decile [3] as used in the other studies. The lowest tertile was classified as low variability, the middle tertile as middle variability, and the highest tertile as high variability.

### Echocardiographic examination

Echocardiographic examinations were performed by a single sonographer who assessed cardiac structural changes and cardiac function. Two-dimensional (2D) echocardiography and M-mode echocardiography were performed using high-resolution ultrasound (Apron EUB-7000HV; Hitachi Medical Corporation, Tokyo, Japan). Left ventricular (LV) diastolic function was assessed by transmittal pulse-wave Doppler to measure mitral inflow early (E) and late (A) diastolic filling velocities, E/A ratio, and tissue Doppler imaging parameters, including the early diastolic mitral annular velocity (e’), which was measured on the lateral side of mitral annulus. The E/e’ ratio was measured as an index of LV filling pressure. Diastolic dysfunction was defined as E/e’ ≥15 [12]. The left atrial dimension was measured on 2D-guided M-mode echocardiographic images of the base of the heart obtained in the parasternal short-axis view. Ejection fraction was measured using a quantitative 2D method (biplane Simpson method of disks) [13]. The LV mass index (LVMI) was calculated using Devereux’s equation and was adjusted for body surface area [14].

Arterial stiffness was assessed by measuring the carotid intima-media thickness (IMT) just after echocardiography by high-resolution ultrasound, as described above, with a 7.5-MHz linear array transducer. The maximum IMT (max-IMT) was defined as the single thickest wall of the near and far right or left walls of the common carotid artery, bulbus, and internal carotid artery, including plaques, as a marker of carotid atherosclerosis [5]. The lesion was scanned bilaterally in longitudinal and transverse projections. The max-IMT was measured at the site of the most advanced atherosclerotic lesion that exhibited the greatest distance between the lumen-intimal interface and the media-adventitia interface. Carotid atherosclerosis was defined as a focal wall thickness of >1.5 mm [15].

### Statistical analyses

Clinical, echocardiography, and cervical ultrasonography variables were compared among the tertile groups of BP variability using analysis of variance for continuous variables or χ2 tests for dichotomous variables. Variables that showed statistically significant differences among the three groups were then included in multivariate analyses. β coefficients (the differences between the highest or middle tertile and the lowest tertile) and 95% confidence intervals (CI) were calculated for each variable by linear regression with adjustment for age (continuous), sex, baseline systolic BP (continuous), use of antihypertensive drugs, and history of CVD. Odds ratios and 95% CIs were calculated for the prevalence of diastolic dysfunction and carotid atherosclerosis by unconditional logistic regression with adjustment for the same covariates. Values of P < 0.05 in adjusted models were considered statistically significant. All analyses were performed using STATA software version 9 (Stata Corp, College Station, TX, USA).

## Results

BP was measured for 9.5 ± 2.9 times during one year (range 4 to 13 times), and mean systolic BP was 130 ± 10 mmHg; the lowest tertile (SD <8.29 mmHg), the middle tertile (SD 8.29-10.99 mmHg), and the highest tertile (SD >10.99 mmHg). The clinical, echocardiography, and cervical ultrasonography variables for the study subjects are summarized by BP variability level in Tables 1 and 2. The high BP variability group tended to be older, had a higher proportion of female subjects, and had higher lipid levels compared with the other groups of subjects, although the differences in these are not statistically significant. The mean ejection fraction was 57%; two-thirds of the subjects had normal systolic function (ejection fraction ≥55%). The LVMI was greatest in the high BP variability group, although this difference was not statistically significant. There were no differences in ejection fraction among the three groups.

**Table 1:** **Characteristics of subjects according to the level of blood pressure variability (n = 144)**

	Blood pressure variability			
	Low variability	Middle variability	High variability	P value
n	48	48	48	
Age (years)	72.3 ± 9.1	70.8 ± 8.5	74.7 ± 8.7	0.101^a^
Male	24 (50.0%)	21 (43.8%)	19 (39.6%)	0.586^b^
Blood pressure measurements (times)	9.1 ± 3.1	9.5 ± 2.6	9.7 ± 2.8	0.569^a^
Systolic blood pressure (mmHg)	130.2 ± 10.1	132.0 ± 12.2	131.5 ± 16.9	0.801^a^
Diastolic blood pressure (mmHg)	72.1 ± 11.0	72.8 ± 11.8	71.2 ± 10.3	0.780^a^
Pulse pressure (mmHg)	58.1 ± 12.3	59.2 ± 11.5	60.3 ± 15.4	0.728^a^
Heart rate (beats/min)	73.8 ± 9.8	70.5 ± 6.8	72.3 ± 6.9	0.248^a^
Antihypertensive medication	33 (68.8%)	38 (79.2%)	41 (85.4%)	0.140^b^
Calcium-channel blocker	27 (56.3%)	30 (62.5%)	34 (70.8%)	0.331^b^
Renin-angiotensin antagonist	20 (41.7%)	19 (39.6%)	24 (50.0%)	0.553^b^
β-blocker	6 (12.5%)	9 (18.8%)	3 (6.3%)	0.180^b^
Fasting plasma glucose (mg/dL)	116.3 ± 41.5	118.8 ± 47.8	115.5 ± 38.9	0.923^a^
HbA1c (%)	6.12 ± 0.94	5.98 ± 0.80	5.82 ± 0.60	0.173^a^
Glucose-lowering medication	8 (16.7%)	8 (16.7%)	8(16.7%)	1.000^b^
LDL-cholesterol (mg/dl)	114.2 ± 24.0	118.0 ± 28.2	124.6 ± 25.5	0.146^a^
Lipid-lowering medication	13 (27.1%)	16 (33.3%)	17 (35.4%)	0.660^b^
Uric acid (mg/dL)	5.50 ± 1.26	5.25 ± 1.29	5.38 ± 1.32	0.643^a^
Body mass index (kg/m^2^)	24.13 ± 3.87	24.03 ± 3.20	24.19 ± 3.56	0.977^a^
eGFR (ml/min/1.73 m^2^)	68.0 ± 19.4	67.5 ± 15.9	64.9 ± 17.8	0.667^a^
Proteinuria	4 (8.3%)	6 (12.5%)	7 (14.6%)	0.627^b^
Cardiovascular disease	9 (18.8%)	8 (16.7%)	7 (14.6%)	0.861^b^

Data are means ± standard deviation or n (%). Low, middle, and high variability are defined as the lowest (<8.29 mmHg), middle (8.29–10.99 mmHg), and highest (>10.99 mmHg) tertiles of the standard deviation of systolic blood pressure, respectively.

LDL-cholesterol, low-density lipoprotein cholesterol; eGFR, estimated glomerular filtration rate.

^a^Analysis of covariance; ^b^χ^2^ test.

**Table 2:** **Echocardiography, and cervical ultrasonography characteristics of subjects according to the level of blood pressure variability (n = 144)**

	Blood pressure variability			
	Low variability	Middle variability	High variability	P value ^a^
n	48	48	48	
Left atrium diameter (mm)	37.3 ± 5.0	36.4 ± 4.6	37.6 ± 6.2	0.541
E (m/s)	0.53 ± 0.13	0.59 ± 0.19	0.57 ± 0.20	0.162
A (m/s)	0.71 ± 0.15	0.77 ± 0.17	0.77 ± 0.19	0.153
e’ (m/s)	0.054 ± 0.019	0.053 ± 0.012	0.048 ± 0.019	0.189
E/A	0.77 ± 0.26	0.80 ± 0.29	0.76 ± 0.29	0.845
E/e’	10.66 ± 3.30	11.60 ± 3.36	13.03 ± 5.33	0.019
LVMI (mm)	139.0 ± 27.3	137.0 ± 23.6	148.1 ± 30.7	0.108
Ejection fraction (%)	58.3 ± 9.1	58.2 ± 6.8	57.5 ± 8.1	0.859
Max-IMT (mm)	1.42 ± 0.46	1.36 ± 0.37	1.65 ± 0.43	0.003

Data are means ± standard deviation or n (%). Low, middle, and high variability are defined as the lowest (<8.29 mmHg), middle (8.29–10.99 mmHg), and highest (>10.99 mmHg) tertiles of the standard deviation of systolic blood pressure, respectively.

E, early ventricular filling velocity; A, late ventricular filling velocity; e’, early diastolic mitral annular velocity; LVMI, left ventricular mass index; max-IMT, maximum intima-media thickness.

^a^Analysis of covariance.

Because E/e’ and max-IMT were significantly different between the BP variability groups (P <0.05 by Tukey method), multivariate analyses were performed to examine the associations between BP variability and E/e’ and max-IMT. Both E/e’ and max-IMT were significantly greater in the high BP variability group than in the low BP variability group also in the multivariate models (high BP variability, middle BP variability vs. low BP variability; E/e’: 13.03 ± 5.33, 11.60 ± 3.36 vs. 10.66 ± 3.30, multivariate-adjusted difference (β) = 1.82, 95% CI 0.06–3.58 for high BP variability and β = 0.92, 95% CI −0.44–2.29 for middle BP variability; max-IMT 1.65 ± 0.43 mm, 1.36 ± 0.37 mm vs. 1.42 ± 0.46 mm, β = 0.20 mm, 95% CI 0.03–0.36 mm for high BP variability and β = −0.02 mm, 95% CI −0.17–0.13 mm for middle BP variability).

Additionally, the prevalence rates of diastolic dysfunction and carotid atherosclerosis were greater in the high BP variability group than in the low BP variability group. The risk of carotid atherosclerosis was significantly greater in the high BP variability group compared with the low variability group (69% vs. 38%, OR 4.93, 95% CI 1.75–13.91) in the multivariate model. Also, the risk of diastolic dysfunction was greater in the high BP variability group compared with the low variability group although it was not statistically significant after adjustment (29% vs. 10%; OR 2.71, 95% CI 0.78–9.38) (Table 3).

**Table 3:** **Prevalence of diastolic dysfunction and carotid atherosclerosis according to the level of blood pressure variabilit**

	Blood pressure variability							
	Low variability		Middle variability			High variability		
				Crude OR	Adjusted OR ^a^		Crude OR	Adjusted OR ^a^
	n (%)	OR	n (%)	(95% CI)	(95% CI) ^a^	n (%)	(95% CI)	(95% CI) ^a^
Diastolic dysfunction								
E/e’ <15	43 (89.6)		41 (85.4)			34 (70.8)		
E/e’ ≥15	5 (10.4)	1 (reference)	7 (14.6)	1.47 (0.43–5.00)	1.33 (0.36–4.92)	14 (29.2)	3.54 (1.16-10.81)	2.71 (0.78–9.38)
Carotid atherosclerosis								
Max-IMT ≤1.5 mm	30 (62.5)		29 (60.4)			15 (31.2)		
Max-IMT >1.5 mm	18 (37.5)	1 (reference)	19 (39.6)	1.09 (0.48–2.48)	1.47 (0.53–4.08)	33 (68.8)	3.67 (1.58-8.54)	4.93 (1.75–13.91)

OR, odds ratio; CI, confidence interval; E/e’, early ventricular filling velocity/early diastolic mitral annular velocity; IMT, intima–media thickness.

^a^Adjusted for age, sex, baseline systolic blood pressure, use of antihypertensive drugs, and history of cardiovascular disease.

## Discussion

To the best of our knowledge, this was the first study to reveal that greater visit-to-visit BP variability is significantly associated with both decreased diastolic function and carotid atherosclerosis independently of mean BP. These findings may provide new insights into the mechanism underlying the relationship between visit-to-visit BP variability and CVD.

Recent data suggest that BP variability is a strong prognostic factor for stroke, coronary heart disease, and all-cause mortality [1–4]. There are several methods to measure BP variability; visit-to-visit BP variability represents long-term BP variability while 24-h BP variability assessed by ambulatory blood pressure monitoring represents short-term BP variability. Prior studies showed that greater visit-to-visit BP variability was independently associated with increased risk of coronary heart disease [4], stroke [3], and all-cause mortality [2]. Visit-to-visit BP variability may be a predictor of CVD compared with mean BP [3] or 24-h BP variability [16]. Thus, visit-to-visit BP variability is a useful and easily measurable marker of CVD.

This study showed that visit-to-visit BP variability was associated with carotid IMT. Carotid IMT is a well-established marker of subclinical atherosclerosis, and it is strongly associated with the risk of future CVD events including coronary heart disease and stroke [5]. Recent studies have demonstrated that visit-to-visit BP variability is also associated with aortic distensibility [9] and endothelial dysfunction [10], which are also markers of subclinical atherosclerosis [17, 18]. Higher 24-h BP variability was reported to be a strong predictor of IMT increase [19], and one study demonstrated an association between higher visit-to-visit BP variability and greater IMT [6]. Fluctuations in BP cause a vascular damage [11]. Steep BP variations increase the oscillatory shear stress in the vessel wall of medium and large arteries. This enhances the traumatic effect of intravascular pressures on the vessel wall that ultimately results in the progression of atherosclerosis [20, 21]. Meanwhile impairments in baroreflex sensitivity caused by structural changes of blood vessels increases BP variability [22]. These mechanisms may explain the relationship between BP variability and IMT.

Diastolic dysfunction is believed to be the main cause of heart failure with preserved ejection fraction [23]. Previous studies revealed that diastolic dysfunction was a predictor of CVD mortality [24], and had a prognostic significance similar to systolic dysfunction [25]. Little is known about the association between BP variability and diastolic function. To our knowledge, only one study of 40 hypertensive patients has revealed an association between visit-to-visit BP variability and diastolic function [8]. This relationship might be mediated by impaired baroreflex sensitivity. Impaired baroreflex sensitivity caused by sinoaortic denervation resulted in an increase in BP variability and diastolic dysfunction in an animal model [26, 27], which suggests that sympathetic modulation of BP can cause diastolic dysfunction. Another mechanism might involve the association between arterial stiffening and diastolic dysfunction (that is, arterial–ventricular coupling) [28, 29], which was reported to cause heart failure with preserved ejection fraction [30, 31]. Because cardiac relaxation is delayed if cardiac afterload is increased by arterial stiffening [28], some studies have shown that greater carotid IMT is associated with diastolic dysfunction [32, 33].

The LVMI was greater in subjects with higher BP variability, which was consistent with the former study [34], although it was not statistically significant. And systolic function was not associated with BP variability. Diastolic dysfunction precedes LV hypertrophy in the development of hypertension [35], and diastolic dysfunction precedes systolic dysfunction in the process subclinical atherosclerosis [33]. Thus, the association between BP variability and diastolic function may precede the development of LV hypertrophy and systolic dysfunction.

Several limitations of this study should be mentioned. First, because of the relatively small number of subjects, we could not perform analyses after stratifying the subjects according to their age or the presence of hypertension. Most of the prior studies that showed associations between BP variability and diastolic function or IMT were performed in elderly subjects with hypertension [6, 7], and the effects of BP variability might be differ between age groups or sexes [36]. Further studies are needed to examine whether the associations between BP variability and diastolic function or IMT are also apparent in middle-aged subjects or non-hypertensive subjects. Also, the study with a larger sample size is required to confirm the result of the multivariable adjustments. The strength of the association became weaker for diastolic dysfunction and stronger for carotid atherosclerosis after adjustment, which should be considered in the future studies. Second, the number of visits to assess BP variability was rather small, the number of visits was not the same for all the subjects in this study, and the interval between visits were not the same, which influence BP variability [37]. The study with a larger number of visits and the same number of visits for all the subjects is needed for the better assessment of BP variability, especially in the study with small number of subjects. Third, these data were collected during routine care and were extracted from medical charts, thus the patients for the examinations were not randomly collected for this study, which may affect the study results. Fourth, considering the cross-sectional design of this study, we could not determine whether treating patients with high BP variability can prevent the progression to diastolic dysfunction and carotid atherosclerosis. This possibility should be examined in longitudinal studies.

## Conclusions

Visit-to-visit BP variability recorded over 1 year was associated with cardiac diastolic function and carotid atherosclerosis. In addition to other well-defined risk factors, we suggest that visit-to-visit BP variability is considered as a marker for the development of diastolic dysfunction and atherosclerosis. Measurements of cardiac function and carotid atherosclerosis may be warranted in subjects with high BP variability.
